# Desiccation as a Post-maturation Treatment Helps Complete Maturation of Norway Spruce Somatic Embryos: Carbohydrates, Phytohormones and Proteomic Status

**DOI:** 10.3389/fpls.2022.823617

**Published:** 2022-02-14

**Authors:** Kateřina Eliášová, Hana Konrádová, Petre I. Dobrev, Václav Motyka, Anne-Marie Lomenech, Lucie Fischerová, Marie-Anne Lelu-Walter, Zuzana Vondráková, Caroline Teyssier

**Affiliations:** ^1^Institute of Experimental Botany of the Czech Academy of Sciences, Prague, Czechia; ^2^Department of Experimental Plant Biology, Faculty of Science, Charles University, Prague, Czechia; ^3^Plateforme Proteome, University of Bordeaux, Bordeaux, France; ^4^INRAE, ONF, BioForA, Orléans, France

**Keywords:** desiccation tolerance, somatic embryogenesis, phytohormones, *Picea abies* (L.) Karst, proteomics, raffinose family oligosaccharides

## Abstract

Exposure of Norway spruce (*Picea abies*) somatic embryos and those of many other conifers to post-maturation desiccation treatment significantly improves their germination. An integration analysis was conducted to understand the underlying processes induced during the desiccation phase at the molecular level. Carbohydrate, protein and phytohormone assays associated with histological and proteomic studies were performed for the evaluation of markers and actors in this phase. Multivariate comparison of mature somatic embryos with mature desiccated somatic embryos and/or zygotic embryos provided new insights into the processes involved during the desiccation step of somatic embryogenesis. Desiccated embryos were characterized by reduced levels of starch and soluble carbohydrates but elevated levels of raffinose family oligosaccharides. Desiccation treatment decreased the content of abscisic acid and its derivatives but increased total auxins and cytokinins. The content of phytohormones in dry zygotic embryos was lower than in somatic embryos, but their profile was mostly analogous, apart from differences in cytokinin profiles. The biological processes “Acquisition of desiccation tolerance”, “Response to stimulus”, “Response to stress” and “Stored energy” were activated in both the desiccated somatic embryos and zygotic embryos when compared to the proteome of mature somatic embryos before desiccation. Based on the specific biochemical changes of important constituents (abscisic acid, raffinose, stachyose, LEA proteins and cruciferins) induced by the desiccation treatment and observed similarities between somatic and zygotic *P. abies* embryos, we concluded that the somatic embryos approximated to a state of desiccation tolerance. This physiological change could be responsible for the reorientation of Norway spruce somatic embryos toward a stage suitable for germination.

## Introduction

Somatic embryogenesis could play an important role in the commercial breeding of conifers, but many problems have to be solved before its widespread use in forest management, e.g., insufficient quality and yield of mature embryos and their low ability to germinate (Pullman and Bucalo, 2014). Eliminating these obstacles would enable enhanced embryogenic culture production, e.g., large-scale cultivation in bioreactors, which offers a promising method for conifer somatic embryogenesis owing to its increased efficiency and lower costs (Välimäki et al., 2020).

Desiccation of somatic embryos (SEs) prior to germination is a possible approach to enhance the amount and quality of germinated embryos. SEs of many conifer species are able to germinate without a prior desiccation phase as desiccation demand is species and/or genotype-specific (Klimaszewska et al., 2016). Nevertheless, mild or partial drying of SEs of *Picea* sp. has been suggested as a prerequisite for successful germination (e.g., Roberts, 1991; Find, 1997; Pond et al., 2002). Moreover, desiccation not only stimulates germination of SEs but also positively influences their conversion into plantlets. However, this effect depends strongly on the length and intensity of desiccation treatment. Slow long-term partial desiccation (up to 5 weeks) under high relative humidity may be more beneficial (Roberts et al., 1991) than 2-week desiccation under a lower relative humidity < 80% (Roberts et al., 1990b; Attree et al., 1991). Subjecting desiccation-sensitive cells to desiccation conditions of < 90% relative humidity can cause the loss of correct protein conformation, membrane phase transitions and RNA/DNA structural rearrangements and fragmentation (Leprince and Buitink, 2015).

The process of conifer embryogenesis is controlled by phytohormones. Their role in individual steps of somatic embryogenesis differs according to the developmental processes taking place in embryonic cultures or SEs. Endogenous levels of phytohormones in embryos are also affected by the hormonal composition of the cultivation medium. Auxins and cytokinins (CKs) are typical components of induction and proliferation media promoting cell division and growth, whereas abscisic acid (ABA) is added to culture media during the maturation step. ABA induces the maturation of conifer SEs, inhibits precocious germination during maturation, promotes the accumulation of storage proteins (Roberts et al., 1990a; Leal et al., 1995; von Aderkas et al., 2002) and contributes to the acquisition of desiccation tolerance (Hoekstra et al., 2001). High ABA levels have been shown to inhibit germination after maturation of hybrid larch SEs (Lelu and Label, 1994). However, the germination rate of hybrid larch SEs was improved by a period of partial drying in the cell under controlled relative humidity (Lelu et al., 1995). An extended desiccation phase was also shown to be required by Sitka spruce SEs when maturing on medium with high ABA content (Roberts et al., 1991). The positive effect of desiccation on the number and quality of germinated SEs has been attributed to decreased ABA levels (Dronne et al., 1997; Find, 1997) but also changes in carbohydrate and storage protein content (Bomal et al., 2002; Wang et al., 2014) and activation of antioxidant systems.

The development of conifer SEs encompasses key stages of zygotic embryogenesis in seeds (Attree and Fowke, 1993; Hakman, 1993; Nagmani et al., 1995; von Arnold et al., 2019). Mimicking conditions during seed development in an *in vitro* somatic embryogenesis system could enable optimization of cultivation procedures (Pullman and Bucalo, 2014). The late maturation phase in orthodox seeds is a period when seeds, after the accumulation of storage reserves, lose water and change to a quiescent dry state. Even though the term desiccation evokes the physical process of drying, this phase involves more than just water loss owing to physiological and biochemical changes taking place in seeds (Angelovici et al., 2010; Leprince et al., 2017). Acquisition of tolerance to certain stresses (low temperatures, water shortages and desiccation) during the final maturation phase is of special importance. This step must prepare seeds to not only withstand the loss of water but also to perform the synthesis of transcripts and proteins necessary for seed germination. These include proteins involved in protection against oxidative stress during dehydration and subsequent rehydration, meristematic growth, or provision of energy supply (Stasolla et al., 2004a; Lara-Chavez et al., 2012; Jing et al., 2017). Synthesis of protective substances, often starting during maturation, is amplified during the acquisition of tolerance to desiccation. Several families of proteins [e.g., LEA and small heat shock proteins (HSPs)] participate in this process by protecting the membrane structures of cells against oxidative species produced during desiccation (Huang et al., 2012; Amara et al., 2014). Their synthesis is also amplified in conifer SEs during late maturation or a desiccation phase (Stasolla et al., 2004b; Jing et al., 2017).

Proteins that provide enzymatic, structural or energetic functions are also important actors in the embryogenesis process. The energetic function is achieved by storage proteins, which also provide nitrogen during germination for *de novo* protein synthesis. Proteins that mainly accumulate during the end of maturation (Leprince and Buitink, 2010) are vicilin- and legumin-like proteins (Flinn et al., 1993; Klimaszewska et al., 2004), although albumin may also participate (Morel et al., 2014). In addition to these specific proteins, accumulation of raffinose family oligosaccharides (RFOs) and sucrose is considered a marker of the late maturation phase owing to their protective functions and is related to the acquisition of desiccation tolerance (Hoekstra et al., 2001; Leprince et al., 2017). Non-structural carbohydrates in plants are multifunctional compounds that may offer a method for improving embryogenesis. Their widely accepted roles include a source of energy and carbon skeleton, osmotic agents, protection of the plant cell ultrastructure, direct signaling role and regulation of gene expression. A pattern of carbohydrate accumulation during the late maturation of zygotic embryos (ZEs) has been reported in conifer species such as *Picea abies* (Gösslová et al., 2001), *Pinus pinaster* (Morel et al., 2014) and *Pinus taeda* (Pullman and Buchanan, 2008). The prominent role of carbohydrates, including their exogenous supply, during conifer embryogenesis *in vitro*, is known (Lipavská and Konrádová, 2004). The distinct patterns of carbohydrate content and spectra during maturation, desiccation and germination have been suggested as indicators of regular Norway spruce SE development (Businge et al., 2013; Hudec et al., 2016). The importance of the RFO: sucrose (R/S) ratio for desiccation protection has also been reported (Sauter and van Cleve, 1991; Xiao and Koster, 2001). In maritime pine ZEs, the R/S ratio was shown to gradually increase during the late phase of maturation and the start of drying (Morel et al., 2014).

We have previously demonstrated (Vondráková et al., 2013) the positive effect of desiccation treatment on the germination capacity of mature Norway spruce SEs. To understand the molecular mechanisms involved in this process, in the present study, we investigated the histological and biochemical changes that occur in Norway spruce SEs after exposure to a post-maturation desiccation treatment. Phytohormone, carbohydrate and protein profiles were determined together with proteomic analysis of molecular functions and activated biological processes. Data were compared with that of ZEs.

## Materials and Methods

### Plant Material

An embryogenic culture of *Picea abies* (L.) H. Karst, genotype AFO 541, was induced at AFOCEL (Nangis, France) from immature ZEs and received as a generous gift from Dr. Bercetche. The embryogenic culture was maintained on solid GD medium supplemented with 2,4-D, BAP and kinetin (Vondrakova et al., 2018). Maturation was performed in Magenta vessels with membrane rafts (Osmotek, Rehovot, Israel) on liquid GD medium (Gupta and Durzan, 1986) at 23 ± 1°C in the dark for five weeks and was subcultured weekly. The liquid GD medium was supplemented with 20 μM ABA (Sigma-Aldrich), 3.75% polyethylene glycol 4000 (PEG, Sigma-Aldrich) and 30 g L^–1^ of sucrose (Lachema, Brno, Czech Republic); the pH was adjusted to 5.8 before autoclaving. ABA and all organic components, except sucrose, were separately prepared and diluted, filter-sterilized and added to the cooled, autoclaved medium. The PEG solution was autoclaved separately and then added to the medium. After five weeks of maturation, clusters with SEs were grown on the maturation medium for an additional three weeks (subcultured weekly on fresh medium) or selected mature cotyledonary SEs were subjected to desiccation treatment during a three-week desiccation phase. Desiccation of SEs isolated from clusters was carried out under high relative humidity (near 100%) on dry filter paper in small Petri dishes (3 cm in diameter) that were left open and placed in large Petri dishes (18 cm in diameter) containing several layers of filter paper wetted with sterile water to maintain high relative humidity. The large Petri dishes were covered with lids, sealed with parafilm and then incubated in a cultivation room for three weeks under a 12 h photoperiod at 18 ± 1°C (Vondrakova et al., 2018). Throughout this study, we refer to this treatment as “desiccation” and the treated embryos as “desiccated”, even though the water content (WC) in the SEs did not decrease significantly.

Phytohormones, carbohydrates and total protein content were measured in cotyledonary SEs collected after five weeks of maturation (M5) [i.e., in mature SEs according to the standard protocol (Vondrakova et al., 2018)], after seven (M7) and eight (M8) weeks of maturation (i.e., after additional two and three weeks of prolonged cultivation on the maturation medium) and after two (D2) and three weeks (D3) of desiccation. At the same time points, SEs were collected for morphological and histological observations. The maturation samples (M5, M7 and M8) were collected just before subculture. All samples for biochemical analyses were dried on cotton wool, then frozen in liquid nitrogen and stored at -80°C until analysis.

Zygotic embryos (ZEs) were collected from seeds of free-growing, open-pollinated Norway spruce [*Picea abies* (L.) H. Karst] trees in late summer, i.e., August (fresh ZEs; ZEf), and in winter, i.e., December/January (dry ZEs; ZEd). Samples were taken from the mid-part of the cones and processed immediately in the same way as SEs. Only ZEd were used for carbohydrate and phytohormone analyses.

All data were related to the dry weight of the collected material. The dry weight (DW) of all samples was obtained by reduction of the initial moisture content at 105°C to a constant weight (approx. 12 h).

### Histological Analysis

Anatomical and histochemical observations were performed on the embryo sections. Ten embryos were processed for each embedding method and each histochemical staining for each type of sample. For a general overview, embryos were embedded in paraffin, whereas for more detailed cytological observations, embryos were embedded in glycol methacrylate. For embedding in paraffin, embryos were fixed in 50% FAA (5% (v/v) formalin, 5% (v/v) acetic acid, 50% (v/v) ethanol), dehydrated in an ethanol/butanol series, then infiltrated and embedded in paraffin, as described in Vondráková et al. (2015). Longitudinal sections (12 μm thick) were stained with 0.5% Ponceau xylidine in 2% acetic acid (Gutmann et al., 1996) to detect storage proteins (red coloration) or with Lugol’s solution (Sass, 1958) for the detection of starch grains (blue-violet coloration). Both types of histochemical reactions were counterstained with azure II, which colored cellulose cell walls blue. For embedding in glycol methacrylate (Technovit 7100; Heraeus-Kulzer, Werheim, Germany), embryos were fixed in 2.5% glutaraldehyde in 100 mM phosphate buffer, dehydrated in an ethanol series, then infiltrated and embedded according to Eliášová et al. (2017). Longitudinal sections were cut to a thickness of 4-5 μm, stained using the periodic acid-Schiff (PAS) procedure, and counterstained with a solution of 1% amido black 10B in 7% acetic acid (Kong and Yeung, 1992). With the PAS procedure, a pink-red coloration indicated the presence of insoluble carbohydrates (e.g., starch, cellulose), whereas, with the amido black stain, a blue coloration indicated proteins. Paraffin sections were observed under a Jenaval transmission light microscope (Zeiss, Jena, Germany). Images were captured using a Nikon DS-Fi3 camera (Tokyo, Japan) and processed using NIS-Elements AR 5.0 software (Laboratory Imaging, Prague, Czech Republic). Glycol methacrylate sections were observed using an Olympus BX63 light microscope (Tokyo, Japan) equipped with an Olympus DP74 CMOS camera. Images were captured and processed using the cellSens Olympus software.

### Protein Analysis

Soluble proteins were extracted from three biological replicates of samples (about 20 to 50 mg FW (fresh weight) of frozen material, depending on the developmental stage) with 1 ml of buffer containing 2% v/w PVPP, 5% v/v β-mercaptoethanol, 2% v/v SDS, 0.1M DTT, 50 mM Tris HCl (pH 6.8) and 10% (v/v) glycerol. Protein content was determined using the Bradford assay with bovine serum albumin as a standard. Results (mean ± SE of 4 biological repetitions) were expressed as soluble protein content in μg mg^–1^ FW. The same quantities of extracted proteins (15 μg) were separated by sodium dodecyl sulfate-polyacrylamide gel electrophoresis (SDS-PAGE) on a 12% gel with stacking gel (4%) following standard protocols. The gel was stained for proteins with colloidal Coomassie brilliant blue G-250 (CBB-G).

### Carbohydrate Analysis

A procedure described in Hudec et al. (2016) was used for soluble saccharide extraction and determination. Four to six biological replicates of samples of isolated embryos (ca. 70-100 mg FW) were freeze-dried and the dry weight was determined. The material was boiled with 80% (v/v) methanol (0.5 mL) at 75°C for 15 min, the solvent was vacuum evaporated and the residue was resuspended in an appropriate amount of Milli-Q ultrapure water (Millipore). After centrifugation at 14.000 g for 10 min, supernatants were filtered through 0.45 μm membrane filters (Millipore) and analyzed using high-performance liquid chromatography (HPLC) with refractometric detection and a Shodex Sugar SC1011 Pb^2+^ column (Shodex, Tokyo, Japan), mobile phase of Milli-Q water and a flow rate of 0.5 ml min^–1^. The pellets remaining after the extraction of soluble carbohydrates were washed three times with ultrapure water, and then starch was hydrolyzed by α-amylase (Fluka Sigma-Aldrich, St. Louis, USA, 30 U) and amyloglucosidase (Fluka Sigma-Aldrich, St. Louis, USA, 60 U) in Na-acetate buffer (pH 4.5) at 37°C for 8 h. The released glucose was quantified by HPLC. The amount of starch was expressed as the glucose amount after enzymatic cleavage. The average values of all determined carbohydrates together with statistical data are given in Supplementary Table 1.

### Phytohormone Analysis

Analysis of plant hormones at selected time points was conducted as described in Vondrakova et al. (2018). Homogenized samples (ca. 100 mg FW aliquots of three biological replicates for each type of sample) were incubated in cold extraction buffer (methanol/water/formic acid, 15/4/1, v/v/v, -20°C, 500 μL) containing a mixture of stable isotope-labeled internal standards [10 pmol; for details see Vondrakova et al. (2018)]. Two phytohormone fractions were obtained using reversed-phase and ion-exchange chromatography (Oasis-MCX, Waters, Milford, MA, USA): (1) fraction A (eluted with methanol) containing acidic and neutral compounds (auxins, ABA, and their derivatives), and (2) fraction B (eluted with 0.35 M NH_4_OH in 70% methanol) containing hormones of basic character (CKs). Hormonal quantification was performed by a HPLC instrument (Ultimate 3000, Dionex, Sunnyvale, CA, USA) coupled to a hybrid triple quadrupole/linear ion trap mass spectrometer (3200 Q TRAP, Applied Biosystems, Foster City, CA, USA) as described previously (Djilianov et al., 2013), using the isotope dilution method with multilevel calibration curves. All data collected were processed with Analyst 1.5 software (Applied Biosystems), and hormonal concentrations were calculated as the amount per 1 g of dry weight of plant material considering that embryos have different WC at various stages of development. Average values of all determined phytohormones together with statistical data are given in Supplementary Table 2.

### Proteomic Analysis

Total protein extracts were prepared from five replicates for each type of sample (approx. 100 mg FW) as previously described by Teyssier et al. (2014). Briefly, extraction was performed with phenol and precipitation was performed with ammonium acetate in ethanol. Protein pellets were resuspended in 8 M urea, 2 M thiourea, 4% CHAPS buffer adjusted to pH 8.5. Protein concentrations of the samples were determined by the Bradford method.

To limit gel-to-gel variations and to facilitate interpretation of the protein abundance in the two proteome comparisons, we opted for DIGE (differential gel electrophoresis) analysis. Three types of samples, each stained with a different fluorochrome, were loaded simultaneously onto the same gel, thus limiting spot location artifacts. The samples (125 μg each) were labeled with CyDyes™ Fluor minimal dyes (GE Healthcare) Cy2, Cy3 or Cy5 according to the manufacturer’s instructions. The labeled samples were then combined for 2D DIGE analytical gels to include the three different dyes and three different sample types, with a total amount of 300 μg, in each of the five repetition gels. Labeled samples were also used for picking gels of each type of sample, combining all biological repetitions with 100 μg of labeled proteins in the 600 μg total. Both types of mixture (for analytical and picking gels) were loaded onto 24 cm IPG strips, pH 4–7 (Protean IEF Cell system, BioRad, France) to perform the first-dimension separation, whereas 2D polyacrylamide gel electrophoresis (PAGE) was performed with 11% polyacrylamide gels. Low fluorescent glass plates were used for the second dimension to minimize background fluorescence during scanning. Both types of gels were scanned using a Typhoon 9400 Trio variable mode imager (GE Healthcare) using optimal excitation/emission wavelengths for each DIGE fluorochrome (Cy2 498/524 nm; Cy3 554/575 nm; Cy5 648/663 nm) and a resolution of 200 μm. The picking gels were also stained with colloidal CBB-G. In addition to dye image analysis, relative quantification was carried out with PROGENESIS software (Non-linear Dynamics, UK) as described by Teyssier et al. (2014). The criteria for selection after statistical analysis were a threshold fold change of 2 and *P* value < 0.05. The corresponding spots were manually excised from the relevant picking gel and identified by mass spectrometry.

### Protein Identifications by Mass Spectrometry

The destain, wash, proteolysis steps and sample preparation for MS are described elsewhere (Teyssier et al., 2014). Peptide mixtures were analyzed by online capillary nano HPLC (LC Packings, Amsterdam, The Netherlands) coupled to a nanospray LCQ Deca XP ion trap mass spectrometer (Thermo Finnigan, San Jose, CA, USA). Peptides were separated an analytical 75-mm id x 15-cm C18 Pep-Map column (LC Packings) with a 5–40% linear gradient of solvent B in 35 min (solvent A was 0.1% formic acid and solvent B was 0.1% formic acid in 80% ACN) and a 300 nL/min flow rate. MS data were acquired in a positive mode in a data-dependent mode. MS scans were recorded over a m/z range from 300 to 1700). Dynamic exclusion was set to 30 s and top 3 fragmentation in CID mode was performed with a normalized collision energy of 35% and an isolation width of 2 m/z.

Data were searched by SEQUEST through Proteome Discoverer 1.3 (Thermo Fisher Scientific Inc.) against the Whitebuilt36 protein database [67,034 entries (personnel communication by Mark Bohlmann; Lippert et al., 2009)] actualized with pab.aa.fasta realized in 2018 (71158 entries,^1^). Two missed enzyme cleavages were allowed. Mass tolerances in MS and MS/MS were set to 2 Da and 1 Da. Oxidation of methionine and carbamidomethylation on cysteine were searched as a dynamic modification. Peptide validation was performed using the Percolator algorithm (Käll et al., 2007) and only “high confidence” peptides were retained corresponding to a 1% false-positive rate at the peptide level.

### Functional Characterization and Gene Ontology Analysis

Changes in expression relative to appropriate controls were calculated based on the cumulative intensity of each peptide (classifying proteins with ≥ 2-fold change ratios as up- or down-regulated). All sequences were mapped against gene ontology (GO) terms in the *Arabidopsis thaliana* TAIR database^2^ for functional annotation. The proteins were then classified according to their biological functions using Web Gene Ontology Annotation Plot software for biological processes and molecular function (Panther,^3^).

### Statistical Analysis

Statistical analysis of the carbohydrate and phytohormone data was conducted using the Sigma Plot statistical package (Systat Software, San Jose, CA, United States). The significance of differences in mean values was evaluated using a one-way analysis of variance (ANOVA). Mean values were compared using Tukey’s test, and differences between means with *P* < 0.05 were considered significant. Data are shown in Supplementary Tables 1, 2.

For protein analysis, statistical analysis was carried out with R software (version 3.3.2; RStudio, 2021). Effects of the treatments on total protein content were evaluated using one-way ANOVA. Variations of this parameter during maturation were analyzed with multiple comparisons of means with Tukey contrasts (*P* < 0.05). For the 2-D PAGE analysis, the intensity change for each spot was analyzed with Student’s *t*-test based on the normalized spot volume (*P* < 0.05).

To explore and visualize correlations between sample types and measured variables (stachyose, raffinose, sucrose, glucose, fructose, inositol, starch, protein, ABAs, IAAs, CKs, DW/FW), the data were subjected to multivariate analysis with FactoMineR (Lê et al., 2008). The phytohormone content was grouped by families: ABAs pooled ABA, DPA, PA, ABA-GE, NeoPA and 9OH-ABA content; IAAs pooled IAA, IAA-Asp, IAA-Glu, Ox-IAA and PAA content; CKs pooled tZ, tZR, tZROG, tZ9G, tZOG, tZRMP, DHZ, DHZR, DHZROG, DHZ9G, DHZRMP, cZR, cZOG, ZROG, cZ9G, cZRMP, iP, iPR and iPRMP content.

## Results

**FIGURE 1:**
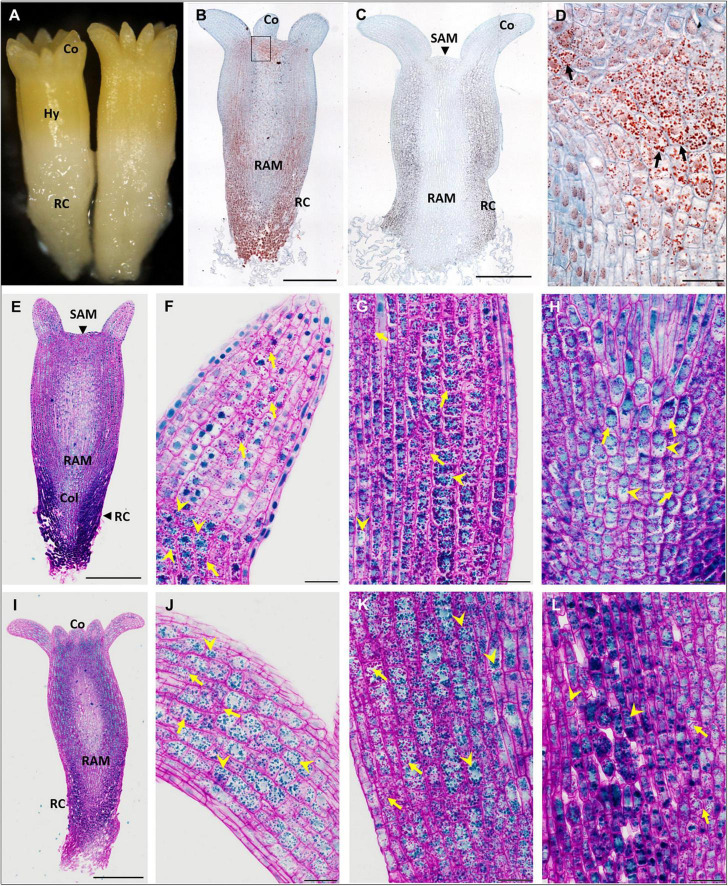
Accumulation of storage compounds in somatic embryos (SEs) of *P. abies* after five and eight weeks of maturation. **(A–H)** Five weeks of maturation (M5); **(I–L)** eight weeks of maturation (M8). **(A)** Morphology of a M5 embryo. **(B)** Longitudinal section of a M5 embryo; storage proteins (in red) were detected in protein storage vacuoles (PSVs) using Ponceau xylidine stain, cell walls were counterstained with azure II (in blue); accumulation of PSVs was prominent below the cotyledons and shoot apical meristem and in the root cap. **(C)** Longitudinal section of a M5 embryo; starch grains were detected using Lugol’s solution (dark points), cell walls were counterstained with azure II; accumulation of starch was prominent mainly in the hypocotyl cortex and root cap, and to a lesser extent under the shoot apical meristem and in the cotyledons. **(D)** Detail of the zone below the cotyledon, corresponding to the inset in panel **(B)**, with prominent PSVs (arrows). **(E)** Longitudinal section of a M5 embryo stained with PAS/amido black (polysaccharides stained magenta in cell walls and starch grains, proteins stained blue in nuclei and PSVs); accumulation of storage proteins was marked in the root cap cells. **(F)** Detail of a cotyledon in panel **(E)** with the predominant accumulation of starch grains (arrows); PSVs accumulated at the bottom of the cotyledon (arrowheads). **(G)** Detail of the hypocotyl cortex in panel **(E)**, where starch grains (arrows) predominated over PSVs (arrowheads). **(H)** Detail of the columella in panel **(E)**, where both storage compounds occurred to a lesser extent than in the root cap cells; starch grains usually surrounded nuclei (arrows); PSVs were more dispersed in the cytoplasm (arrowheads). **(I)** Longitudinal section of a M8 embryo stained with PAS/amido black; accumulation of storage proteins was less prominent in the M8 SE root cap than in M5. **(J)** Detail of the cotyledon in panel **(I)** with abundant PSVs (arrowheads) and starch grains (arrows). **(K)** Detail of the hypocotyl cortex in panel **(I)** with abundant PSVs (arrowheads) and starch grains (arrows). **(L)** Detail of the root cap cells (on the left side) and columella cells (on the right side) in panel **(I)**, both with abundant PSVs (arrowheads) and starch grains (arrows). Co, cotyledons; Col, columella; Hy, hypocotyl; RAM, root apical meristem; RC, root cap; SAM, shoot apical meristem. Scale bars represent: 500 μm in panels **(B,C,E,I)**; 50 μm in panels **(D–L)**.

Mature SEs with well-developed cotyledons, and shoot and root apical meristems after five weeks of maturation (M5) were used as starting material for subsequent experiments (Figure 1A).

**FIGURE 2:**
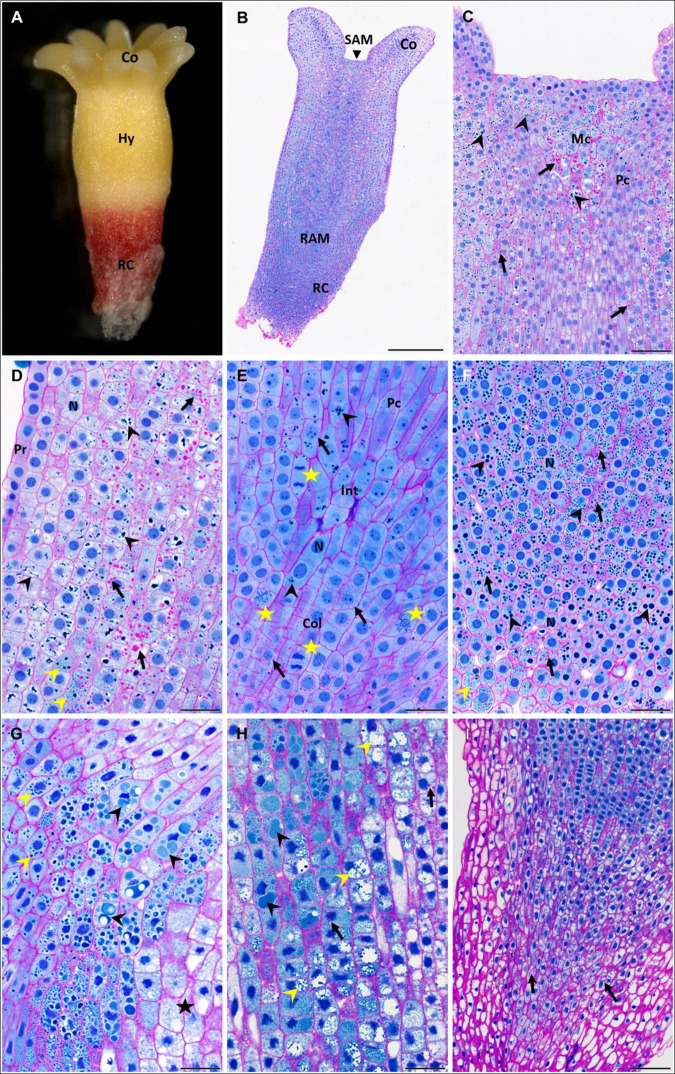
Accumulation of storage compounds in somatic embryos (SEs) of *P. abies* after two and three weeks of desiccation. **(A–F)** Two weeks of desiccation (D2); **(G–I)** three weeks of desiccation (D3). **(A)** Morphology of a D2 embryo; note the red coloring of the root cap that was induced during desiccation in light. **(B)** Longitudinal section of a D2 embryo stained with PAS/amido black [polysaccharides in cell walls and starch grains stained magenta, proteins in nuclei and protein storage vacuoles (PSVs) stained blue]. **(C)** Detail of the shoot apex in panel **(B)**; starch grains (arrows) accumulated frequently in the region of central mother cells of the shoot meristem and in cortical cells but less in cells of the procambium; PSVs (arrowheads) were located similarly. **(D)** Detail of the hypocotyl cortex in panel **(B)**; starch grains occurred predominantly in deeper cortical cells than in sub-protodermal cells (arrows); individual PSVs occurred in the cytoplasm, but more often PSVs clustered and/or fused in vacuoles (black arrowheads); very tiny PSVs occurred in some cells (yellow arrowhead). **(E)** Detail of the root apical meristem in panel **(B)**; starch grains (arrows) often surrounded nuclei; PSVs were rare (arrowheads); note the frequent mitotic figures (stars) next to root initials and in the columella. **(F)** Detail of the root cap in panel **(B)**; starch grains were present in most root cap cells (arrows), but PSVs of different sizes predominated (black arrowheads); very tiny PSVs occurred in the distal region of the embryo (yellow arrowhead). **(G)** Detail of the hypocotyl cortex below the cotyledons of a D3 embryo; starch grains were very rare; intact individual PSVs were observed (yellow arrowheads) in cells located in the central pith of the embryo contrary to cells of the sub-protodermal region (star); in the cells in between, proteinaceous content occurred in large vacuoles as an amorphous material (black arrowheads). **(H)** Detail of the basal part of the hypocotyl cortex of a D3 embryo; starch grains were more abundant than below the cotyledons (arrows); individual PSVs disappeared, while the proteinaceous content occurred in large vacuoles as an amorphous (black arrowheads) or dispersed material (yellow arrowheads). **(I)** Root cap of a D3 embryo was free of PSVs, starch grains occurred only in the central part (arrows). Co, cotyledons; Col, columella; Hy, hypocotyl; Int, root initials; Mc, mother cells of the shoot meristem; N, nucleus; Pc, procambium; Pr, protoderm; RAM, root apical meristem; RC, root cap; SAM, shoot apical meristem. Scale bars represent: 500 μm in panel **(B)**; 100 μm in panels **(C,I)**; 50 μm in panels **(D–H)**.

During maturation prolonged for 2 or 3 weeks, cotyledonary SEs (on the surface of clusters) enlarged. In most of them, disintegration and callogenesis appeared on the surface of the embryos. The isolated SEs grew slowly at the start of desiccation, the root pole turned red (Figure 2A) and later on the cotyledons became green. No morphological changes and no callogenesis occurred during the second and third weeks of desiccation (Figure 2B).

**FIGURE 3:**
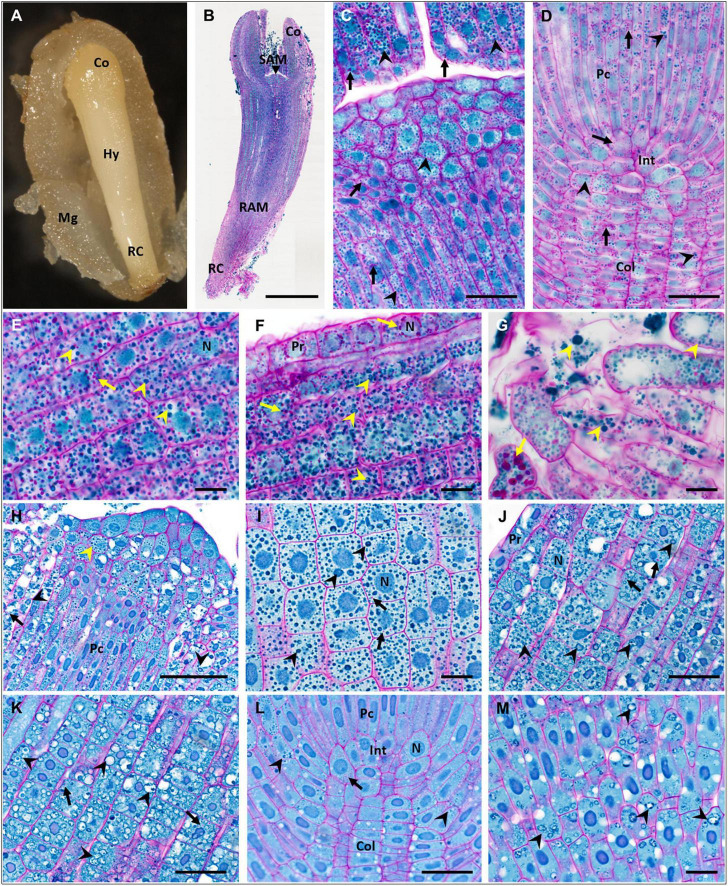
Accumulation of storage compounds in fresh and dry zygotic embryos (ZEs) of *P. abies*. **(A–G)** fresh ZE (ZEf); **(H–M)** dry ZE (ZEd). **(A)** Morphology of a ZEf in a megagametophyte. **(B)** Longitudinal paraffin section of a ZEf stained with PAS/amido black (polysaccharides in cell walls and starch grains stained magenta, proteins in nuclei and protein storage vacuoles (PSVs) stained blue). **(C)** Detail of the shoot apical meristem and part of the cotyledons in panel **(B)**; starch grains (arrows) and PSVs (arrowheads) were abundant in all types of cells. **(D)** Detail of the root apical meristem in panel **(B)**; numerous starch grains (arrows) and PSVs (arrowheads) occurred in all cells of the procambium, root initials and columella. **(E)** Detail of the hypocotyl cortex in panel **(B)** proximal to cotyledons; numerous starch grains (arrows) and intact PSVs (arrowheads) occurred in all cells. **(F)** Detail of protodermal and cortical cells of the basal part of the hypocotyl in panel **(B)**; numerous starch grains (arrows) and PSVs (arrowheads) occurred in all cortical cells; starch grains surrounded nuclei in protoderm. **(G)** Detail of the most distal cells of the root cap in panel **(B)**; starch grains (arrows) and PSVs (arrowheads) of different sizes filled all cells. **(H)** Detail of the shoot apical meristem of a ZEd resin longitudinal section; compact PSVs (yellow arrowhead) occurred in apical and subapical vacuolated cells and in the procambium, whereas in vacuolated cells below cotyledons on both sides of the image, PSVs fused to larger vacuoles with amorphous proteinaceous content (black arrowheads). **(I)** Detail of cells in a ZEd cotyledon; PSVs (arrowheads) predominated over rare tiny starch grains (arrows). **(J)** Detail of protodermal and cortical cells in the apical part of the ZEd hypocotyl; numerous PSVs (arrowheads) of different sizes and proteinaceous content (amorphous or dispersed in fused PSVs) predominated over the rare, tiny starch grains (arrows). **(K)** Detail of cortical cells in the basal part of the ZEd hypocotyl; numerous swollen PSVs (arrowheads) almost free of proteinaceous content predominated over the rare, tiny starch grains (arrows). **(L)** Detail of the root apical meristem of ZEd; only very rare starch grains (arrow) and PSVs (arrowheads) occurred in the cells of the procambium, root initials and columella. **(M)** Detail of the root cap cells of ZEd; only PSVs with dispersed proteinaceous content (arrowheads) were observed. Co, cotyledons; Col, columella; Hy, hypocotyl; Int, root initials; Mg, megagametophyte; N, nucleus; Pc, procambium; Pr, protoderm; RAM, root apical meristem; RC, root cap; SAM, shoot apical meristem. Scale bars: **(B)** 500 μm; **(C,D,J–L)** 50 μm; **(H)** 100 μm; **(E–G,I,M)** 20 μm.

Fresh zygotic embryos collected in late summer were already well morphologically developed, similarly to M5 embryos, and almost filled the corrosion cavity inside the megagametophyte (Figures 3A,B). ZEd collected in winter filled the entire corrosion cavity; morphologically, they differed from ZEf only by more elongated cotyledons.

WC in M5 embryos was 4.5 ± 0.35 g H_2_O g^–1^ DW. During prolonged maturation, WC decreased to 3.6 ± 0.19 g H_2_O g^–1^ DW in M8 embryos. This value was significantly comparable to WC achieved after two weeks of desiccation treatment (3.78 ± 0.44 g H_2_O g^–1^ DW in D2). During the third week of desiccation, WC increased (4.65. ± 0.13 g H_2_O g^–1^ DW) and was not significantly different from the value in M5 embryos. WC in ZEf (0.55 ± 0.08 g H_2_O g^–1^ DW) and ZEd (0.31 ± 0.07 g H_2_O g^–1^ DW) differed significantly from WC values in all SE samples.

During subsequent steps of the investigation, differences between SEs in the development stages M5, M7, M8 and D2, D3 and ZEs were described on biochemical levels, i.e., in the content of non-structural carbohydrates, starch and proteins and in phytohormone profiles. For more precise determination of differences among somatic M5, M7, M8 and D2, D3 embryos and ZEs, histological and proteomic analyses were performed.

### Histological Analysis

The accumulation of storage compounds (proteins and starch) and their localization within maturated (M5, M8) and desiccated (D2, D3) SEs, and within ZEf and ZEd were followed using histological analysis.

In M5 embryos (Figure 1A), storage proteins were deposited in protein storage vacuoles (PSVs) located in the cytoplasm (Figures 1B,E). PSVs accumulated predominantly below the shoot apical meristem (SAM) and at the base of cotyledons (Figures 1B,D,F), in the cortex of the hypocotyl (Figure 1G) and in the root cap (Figures 1B,E). A smaller number of PSVs occurred in the cotyledons (Figure 1F), in the central part of embryos, and in the columella below the root apical meristem (RAM) (Figure 1H). The size of the PSVs differed according to their localization. PSVs accumulated mainly in the upper parts of embryos (cotyledons, upper part of hypocotyl). Toward the root pole of the embryos, the size of PSVs enlarged; the largest ones occurred in cells of the root cap, usually in small numbers and often along with many tiny PSVs. Starch grains were distributed in a similar way as PSVs (Figures 1C,E); they accumulated mainly below the SAM and cotyledons, in the hypocotyl cortex (Figure 1G) and in cells of the root cap (Figure 1C). To a lesser extent, starch grains occurred in the cotyledons (Figure 1F), the central part of embryos, and in the columella (Figure 1H). Generally, the vacuolization of embryo cells was low and limited to cotyledons and/or the very upper region of the hypocotyl. No deposits were observed in the vacuoles. Cell divisions were observed in cells of the protoderm, cotyledons and procambium.

During prolonged maturation, the distribution of storage compounds in SEs did not change (Figure 1I). The accumulation of storage proteins increased greatly in cells of the cotyledons (Figure 1J). PSVs were abundant in both the hypocotyl cortex (Figure 1K) and the root cap (Figure 1L). Numerous starch grains persisted in all parts of the embryos (Figures 1J–L). Cell divisions were not observed.

In desiccated SEs, pronounced changes in the deposition of storage proteins and starch grains (Figure 2B) were found. The vacuolization of cells in the hypocotyl and cotyledons increased during the first two weeks (D2), and vacuoles enlarged further during the third week of desiccation (D3). Numerous PSVs were found inside these vacuoles in the cotyledons and hypocotyl. In the upper part of the hypocotyl below the cotyledons and below the apical meristem (SAM) (Figure 2C), mostly intact PSVs were located in the cytoplasm. In the lower parts of the hypocotyl (toward the root pole) of D2 embryos, mostly intact PSVs occurred in vacuoles (Figure 2D), whereas in D3 embryos, PSVs inside vacuoles exhibited different degrees of degradation (Figures 2G,H). This degradation of PSVs occurred in an ascending gradient toward the root pole. In the lower parts of the hypocotyl, intact PSVs were rare, but the vacuoles contained an amorphous substance that stained blue, which may have been the content of fused PSVs sequestered in the vacuoles (Figures 2G,H). Further toward the root meristem (RAM), PSVs showed a dispersed content (Figure 2H). Vacuolization or degradation/utilization of the PSV content was not observed in the cells of the root cap in D2 (Figures 2E,F), but in D3 root cap cells, almost no PSVs occurred (Figure 2I). Starch grains were found in all parts of the embryo bodies, where they accompanied storage proteins, e.g., below the SAM (Figure 2C), in the hypocotyl (Figure 2D), in the RAM and columella (Figure 2E), and in the root cap (Figure 2F). However, in comparison to M5, M7 and M8 embryos, the amount and size of starch grains decreased markedly during the first two weeks of desiccation. This trend continued during the third week of desiccation. Only rarely, starch grains were found in the hypocotyl (Figures 2G,H). In the root pole, starch grains occurred almost only in the central region of the columella and not in the pericolumn (Figure 2I). Cell divisions occurred in the protoderm, the procambium, below the SAM, in the RAM region (Figure 2E) and occasionally in the cotyledons in D2 embryos and more often in D3 embryos.

Fresh zygotic embryos had abundant PSVs throughout their cotyledons (Figure 3C) and hypocotyl (Figures 3E,F), but also in the SAM (Figure 3C), in the region of the RAM, procambium and columella (Figure 3D), and in cells of the root cap (Figure 3G). Numerous starch grains were found in all these tissues (Figures 3C–G). Numerous PSVs were also found in ZEd, e.g., in the SAM (Figure 3H) and cotyledons (Figure 3I), and tiny PSVs occurred in the columella (Figure 3J). In the hypocotyl, cells were highly vacuolated. PSVs merged in the upper part of the hypocotyl (toward the cotyledons) (Figure 3K) and were probably utilized. In the lower part of the hypocotyl, a higher extent of utilization of storage proteins was found (Figure 3L). Utilization of storage proteins also occurred in cells of the root cap (Figure 3M). Small starch grains were rarely found in the cotyledons (Figure 3I), hypocotyl (Figures 3K,L) and columella (Figure 3J).

### Protein Analysis

**FIGURE 4:**
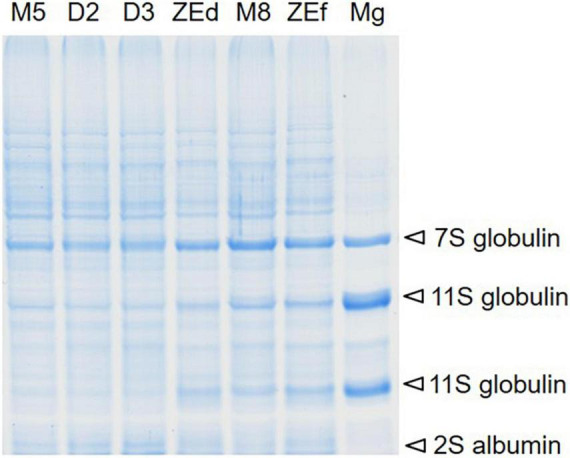
Representative SDS-PAGE total protein profile comparison in somatic (SEs) and zygotic (ZEs) embryos of *P. abies.* The location of major storage proteins identified by mass spectrometry in SEs, dry ZEs and the megagametophyte (Mg) in the protein pattern is shown by arrows. Line M5 – SEs after 5 weeks of maturation, line M8 – SEs after 8 weeks of prolonged maturation, line D2 – SEs after 2 weeks of desiccation, line D3 – SEs after 3 weeks of desiccation, line ZEd – dry ZE, ZEf – fresh ZE, Mg – megagametophyte.

No change in protein content was found when SEs were kept on the maturation medium during prolonged maturation (M5 versus M7 and M8 embryos). Desiccation induced a decrease in protein content in D2 and D3 embryos in comparison to M5 embryos (Table 1). The protein content in the ZEs was higher than in all stages of SEs. The treatment effect was significant with a *P* value < 2.05 × 10^–06^. Electrophoresis profile analysis of total protein extracts was performed as a qualitative assay of SE protein content (Figure 4). Two or three weeks of desiccation did not induce any change in the protein profile in the detected bands and their intensity. In contrast, the intensities of some bands increased in ZEs as well as in M8 embryos. These bands corresponded to storage proteins identified by mass spectrometry as cruciferina (also called 7S-globulin), cruciferin (11S-globulin) and 2S albumin. They also corresponded to the major bands in the megagametophyte.

**TABLE 1:** Quantitative analysis of total proteins in somatic embryos after five weeks of maturation (M5), two (M7) and three weeks (M8) of prolonged maturation, and after two (D2) and three (D3) weeks of desiccation compared to cotyledonary fresh ZEs.

Samples	Total protein content
	(μg.mg-1 FW)
M5	126.0 ± 4.9^ab^
M7	114.2 ± 13.0^a^
M8	111.4 ± 16^a^
D2	96.8 ± 7.5^a^
D3	94.0 ± 4.3^a^
ZE	146.0 ± 7.0^b^

*Values are means of three biological repetitions ± SE. Significant differences at P < 0.05 are indicated in different letters.*

### Non-structural Carbohydrate Analysis

**FIGURE 5:**
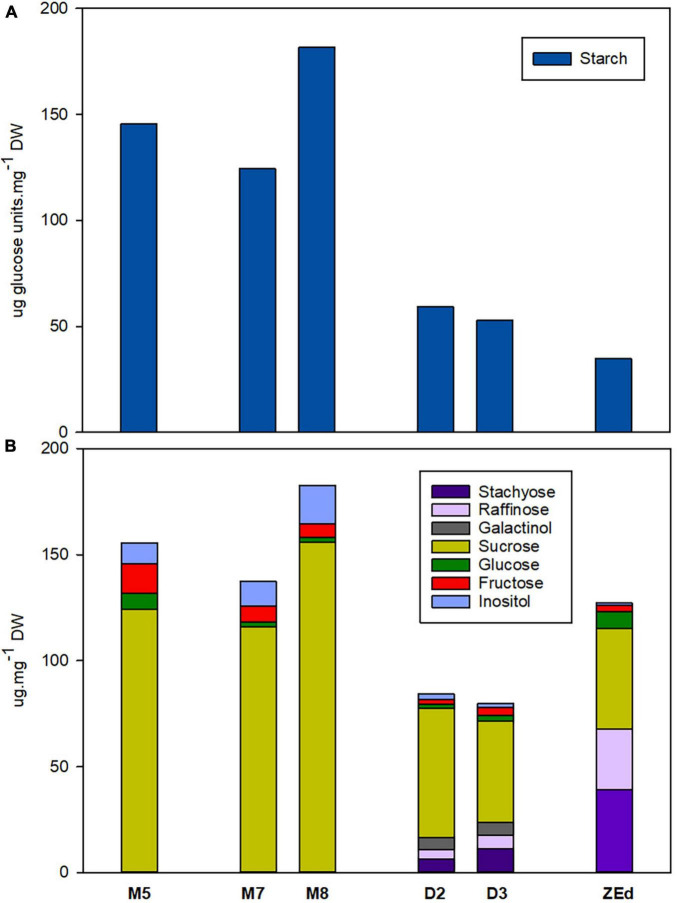
Endogenous non-structural carbohydrates in *P. abies* somatic (SEs) and zygotic (ZEs) embryos. **(A)** Starch content (expressed as glucose amount after enzymatic starch cleavage); **(B)** Soluble carbohydrate content and spectrum. M5 – mature SEs after 5 weeks of maturation, M7 and M8 – SEs after 7 and 8 weeks of prolonged maturation, D2 and D3 – SEs after 2 and 3 weeks of desiccation, ZEd – dry ZEs; *n* = 4–6. Components of carbohydrate spectrum that accounted for less than 1% of the total carbohydrates are included only in Supplementary Table 1.

During prolonged maturation, starch levels in SEs remained high. The starch content in M7 embryos was comparable to that in M5 embryos but increased significantly a week later (M8). However, the starch content differed significantly in desiccated embryos. Desiccation treatment led to a marked decrease (to about 1/3 of the value of M5 embryos) of starch content compared to that detected in D2 and D3. Similarly low content of starch was found in ZEd (Figure 5A).

M5 embryos contained high amounts of sucrose together with low content of hexoses (with fructose being more abundant than glucose). Prolonged maturation did not lead to significant changes in the carbohydrate spectrum, which exhibited only a slight increase in total soluble carbohydrates (M8). This was in sharp contrast to D2 and D3, where the total carbohydrate content was reduced. The proportion of monosaccharides was very low compared to the proportion of disaccharide sucrose. In D2 and D3, newly emerged RFOs, i.e., raffinose and stachyose, and galactinol (an intermediate of RFO synthesis) were detected. In D3 embryos, RFOs represented more than 23% of the total soluble carbohydrates and the proportion of RFOs to sucrose (R/S ratio) was 0.37. Sucrose contributed the main part of the spectrum in all SE samples under study. Embryos also contained inositol in their carbohydrate spectra (Figure 5B).

The corresponding characteristics were followed in fully developed isolated ZEd. The soluble saccharide spectrum comprised RFOs and sucrose as the prevailing constituents (together accounting for approx. 92% of total soluble saccharides, R/S ratio = 1.43), and hexoses (with glucose being more abundant than fructose) and inositol as minor constituents (Figure 5B). Traces of galactose were identified in the spectrum of ZEd (see Supplementary Table 1).

### Phytohormone Analysis

#### Abscisic Acid and Its Derivatives

M5 embryos contained relatively high levels of ABA and its derivatives (approx. 630 000 pmol/g DW), which reflected the addition of exogenous ABA to the liquid maturation medium. Despite that, a significant reduction of endogenous ABA content in embryos occurred during prolonged maturation. After three weeks of prolonged maturation (M8), levels of ABA reached 51% of the concentration detected in M5 embryos. The content of ABA derivatives was relatively low (in comparison to the concentration of ABA itself). DPA, PA and ABA-GE detected in M5 embryos were also found after prolonged maturation. Compared to M5 embryos, DPA and PA levels decreased significantly during prolonged maturation (M8) to 66 and 58%, respectively. In contrast, a significant increase in ABA-GE content was recorded in M8. An even greater decrease in ABA levels was detected in desiccated embryos. At the end of desiccation (D3), levels of ABA had dropped significantly to 27% of its M5 concentration. PA, DPA and other ABA derivatives (NeoPA, 9OH-ABA) were present in minute amounts in desiccated embryos, not exceeding 4% of their respective values in M5. In contrast, the ABA-GE content in embryos at the end of desiccation was comparable to the values found in M5 embryos.

**FIGURE 6:**
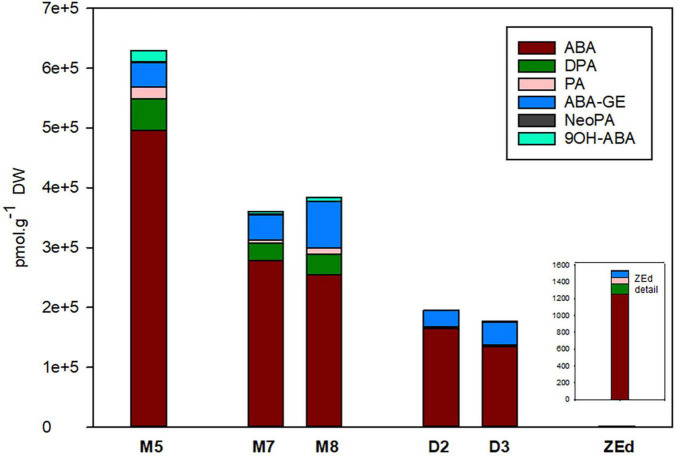
Content of ABA and its derivatives in *P. abies* somatic (SEs) and zygotic (ZEs) embryos. M5 – mature SEs after 5 weeks of maturation, M7 and M8 – SEs after 7 and 8 weeks of prolonged maturation, D2 and D3 – SEs after 2 and 3 weeks of desiccation, ZEd – dry ZEs. ABA, abscisic acid; ABA-GE, ABA-glucosylester; PA, phaseic acid; DPA, dihydrophaseic acid; NeoPA, neophaseic acid; 9OH-ABA, 9-hydroxy-ABA.

In ZEd, the content of ABA and its derivatives was significantly lower than in all analyzed SEs (approx. 1540 pmol/g of DW). Their spectrum was similar to that of M5 embryos, with a predominance of ABA (more than 80%) and lower abundances of DPA (8%), PA (5%) and ABA-GE (5%) (Figure 6).

#### Auxins

The total content of auxins in SEs was relatively low, reaching about 2,000 pmol/g DW in M5 embryos. During prolonged maturation, the total content of auxins in embryos did not change, but the ratio between particular auxin derivatives differed considerably. After three weeks of prolonged maturation (M8), levels of IAA in the embryos decreased significantly to 47% of the value detected in M5 embryos. In contrast, a substantial increase in the concentrations of IAA amino acid conjugates, such as IAA-Asp (262%) and IAA-Glu (237%), was observed in M8. Interestingly, the prolonged maturation did not cause any apparent changes in the levels of Ox-IAA, the major IAA catabolite, and the non-indole phenolic auxin PAA. In desiccated embryos, enhancement of the total auxin content was recorded from the second to the third week of desiccation. This enhancement in D3 was due to considerably higher concentrations of IAA-Asp (216%), Ox-IAA (321%) and PAA (162%) compared to M5 embryos. On the other hand, the IAA content significantly decreased in desiccated embryos (to 80%).

**FIGURE 7:**
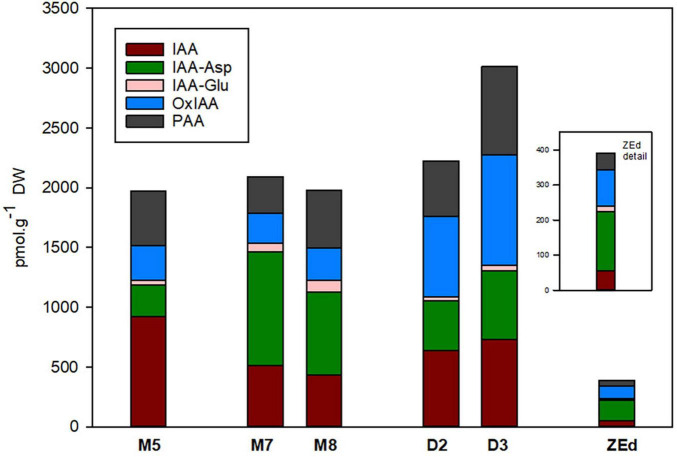
Content of auxins and their derivatives in *P. abies* somatic (SEs) and zygotic (ZEs) embryos. M5 – mature SEs after 5 weeks of maturation, M7 and M8 – SEs after 7 and 8 weeks of prolonged maturation, D2 and D3 – SEs after 2 and 3 weeks of desiccation, ZEd – dry ZEs. IAA, indole-3-acetic acid; IAA-Asp, IAA-aspartate; IAA-Glu, IAA-glutamate; OxIAA, oxo-IAA; PAA, phenylacetic acid.

In ZEd, the concentration of auxins was significantly lower than in SEs (ca. 390 pmol/g DW). The most substantially decreased levels compared to those of M5 embryos were recorded for IAA (17-fold) and PAA (10-fold). In contrast, the decrease was only ca. 1.5 to 3-fold for IAA-Asp and Ox-IAA, respectively, representing the prevailing auxin derivatives in ZEd (Figure 7).

#### Cytokinins

**FIGURE 8:**
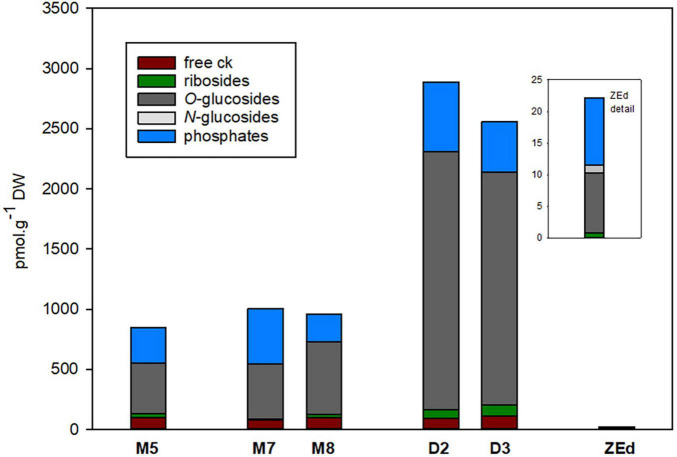
Total content of cytokinin forms in *P. abies* somatic (SEs) and zygotic (ZEs) embryos. M5 – mature SEs after 5 weeks of maturation, M7 and M8 – SEs after 7 and 8 weeks of prolonged maturation, D2 and D3 – SEs after 2 and 3 weeks of desiccation, ZEd – dry ZEs.

The total content of CKs (Figure 8) was relatively low in M5 embryos, not exceeding 850 pmol/g DW. In total, 16 isoprenoid CK derivatives were detected in M5 embryos, including free bases (bioactive forms), ribosides (transport forms), *O-*glucosides (storage forms) and phosphates (immediate biosynthetic precursors). Whereas *O-*glucosides and phosphates represented the predominant forms of CKs (approx. 85% of total CK pool), interestingly, no *N*-glucosides were found in SEs. The content and composition of CKs remained conserved during prolonged maturation. In contrast, a significant increase in CK levels was observed during desiccation. The total CK content in embryos desiccated for 2 and 3 weeks was more than three times higher (approx. 2890 and 2560 pmol/g DW, respectively) compared to M5 embryos. This enhancement was especially caused by an increase (more than 4.5-fold) of *O-*glucoside concentration.

**FIGURE 9:**
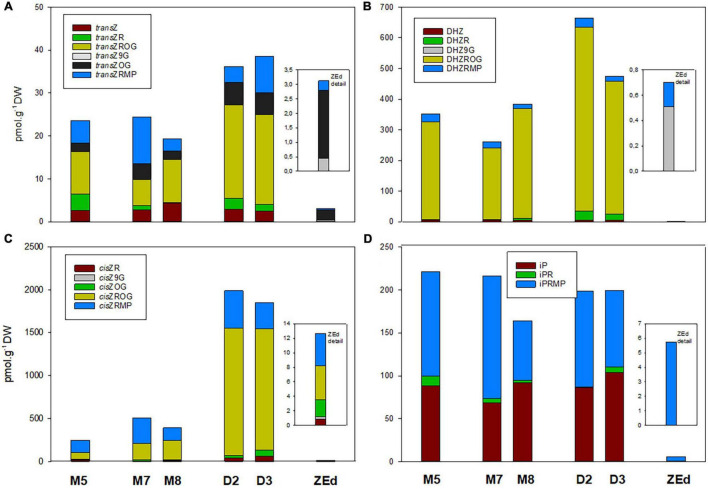
Content of individual cytokinins and their derivatives in *P. abies* somatic (SEs) and zygotic (ZEs) embryos. **(A)** *Trans*-zeatin types: *trans*Z, *trans*-zeatin; *trans*ZR, *trans*-zeatin 9-riboside; *trans*ZOG, *trans*-zeatin *O*-glucoside; *trans*ZROG, *trans*-zeatin 9-riboside *O*-glucoside; *transZRMP, trans*-zeatin 9-riboside-5′-monophosphate. **(B)** Dihydrozeatin types: DHZ, dihydrozeatin; DHZR, dihydrozeatin 9-riboside; DHZ9G, dihydrozeatin-9-glucoside; DHZROG, dihydrozeatin 9-riboside *O*-glucoside; DHZRMP, dihydrozeatin 9-riboside-5′-monophosphate. **(C)** *Cis*-zeatin types: *cis*ZR, *cis*-zeatin 9-riboside; *cis*ZOG, *cis*-zeatin *O*-glucoside; *cis*Z9G, *cis*-zeatin 9-glucoside; *cis*ZROG, *cis-*zeatin 9-riboside *O*-glucoside; *cis*ZRMP, *cis*-zeatin 9-riboside-5′-monophosphate. **(D)** *N*^6^-(Δ^2^-isopentenyl) adenine types: iP, *N*^6^-(Δ^2^-isopentenyl) adenine**;** iPR, *N*^6^-(Δ^2^-isopentenyl) adenosine; iPRMP, *N*^6^-(Δ^2^-isopentenyl) adenosine-5′-monophosphate. M5 – mature SEs after 5 weeks of maturation, M7 and M8 – SEs after 7 and 8 weeks of prolonged maturation, D2 and D3 – SEs after 2 and 3 weeks of desiccation, ZEd – dry ZEs.

The total CK pool in SEs was composed of *trans*Z (5 forms), *cis*Z, DHZ (4 forms both) and iP (3 forms), and the proportion of each particular group was characterized separately (Figure 9). Generally, DHZ- and *cis*Z-type CKs prevailed in M5 embryos as well as during prolonged maturation, with concentrations ranging from 260-380 and 250-505 pmol/g DW, respectively. During desiccation, levels of DHZ- and *cis*Z-types increased substantially compared to those in M5 embryos, primarily due to the enhanced accumulation of DHZROG and *cis*ZROG. Similarly, increased concentrations in desiccated embryos (approx. 40 pmol/g of DW) compared to M5 embryos (approx. 25 pmol/g of DW) were found for *trans*Z-type CKs, representing the least abundant CK forms. In contrast, levels of iP and its derivatives were not much affected by the desiccation step and fluctuated near 200 pmol/g DW in both M5 and desiccated embryos.

The total pool of CKs in ZEd was significantly lower than in SEs (e.g., approx. 38-fold compared to M5 embryos). The CK spectrum was analogous to M5 and desiccated embryos, with *O*-glucosides and phosphates representing the dominant CK forms (42% and 48% of the total CK pool, respectively). Interestingly, in contrast to SEs, *N*-glucosides were detected in ZEd, although in relatively low quantities not exceeding 6% of the total CK pool (Figures 8, 9). Among *N*-glucosides, only those glycosylated at the *N9* but not the *N7* position were found.

### Multivariate Analysis

**FIGURE 10:**
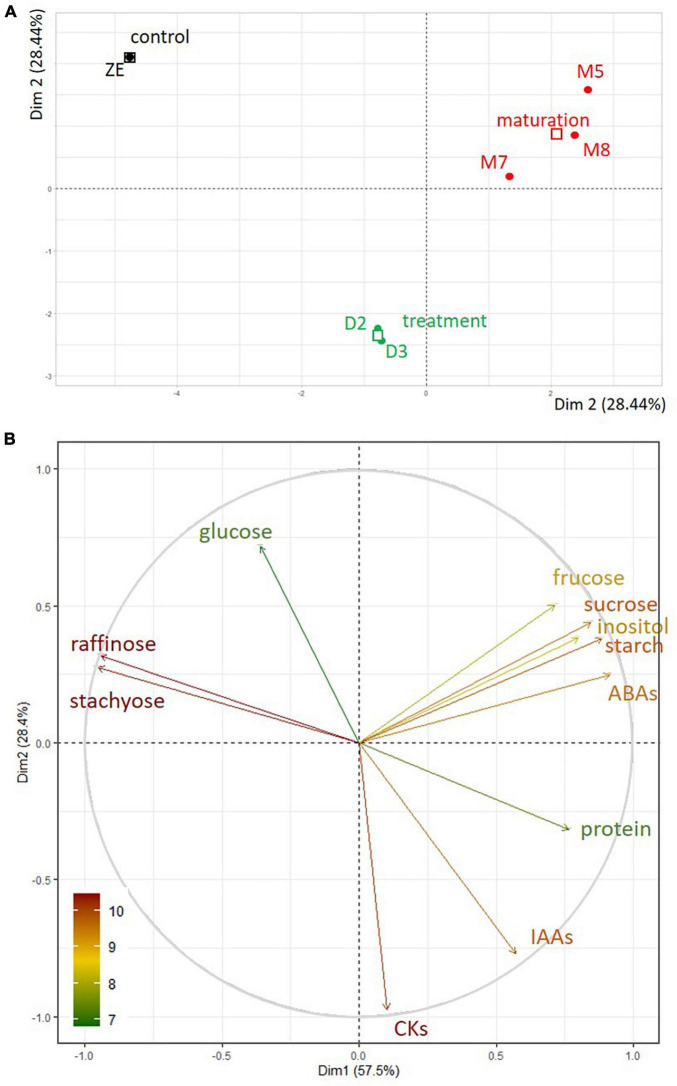
**(A)** PCA of somatic embryos maturing during five (M5) or eight (M8) weeks or desiccated during two (D2) or three (D3) weeks, and dry zygotic embryos (ZE) according to their biochemical dataset (stachyose, raffinose, sucrose, glucose, fructose, inositol, starch, protein, ABAs, IAAs, CKs), in the factorial plan Dim 1 - Dim 2. **(B)** Correlation circle between the biochemical variables in the PCA of the samples in the factorial plan Dim 1 - Dim 2. The contribution of the biochemical variables is represented by the color of their respective vector.

To assess the variation between embryo samples, we performed principal component analysis (PCA) using available biochemical variables (levels of fructose, glucose, inositol, raffinose, stachyose, starch, sucrose, protein, ABAs, IAAs, CKs). The plane formed by the first two main components (Dim 1 and Dim 2, Figure 10A) showed good separation of the samples according to their type. Dim 1 explained almost 60% of the variance between samples. According to the correlation circle (Figure 10B), which represents the correlation between the first two main components and the biochemical characteristics of the samples, the content of ABAs and proteins was strongly correlated with Dim 1, whereas the content of raffinose and stachyose contributed negatively. Whether the correlation was positive or negative, the biochemical variables most involved in this sample type clustering were ABAs, raffinose, stachyose, then proteins. The proximity between the raffinose and stachyose vectors, of equivalent length, indicated a high degree of association between these two variables. CKs and IAAs did not participate in Dim 1 only in Dim 2, which explained 28.1% of the variance between samples. Dim 2 contributed to the separation of the ZE and desiccated SE stages (Figure 10A). The relative and absolute contributions of the biochemical variables to the main components are given in Supplementary Table 3.

### Proteomic Studies

A proteomic study was carried out to (1) define the degree of similarity between M5 embryos and ZEf (comparison M5-ZEf), and (2) to understand the effect of desiccation treatment on protein profile comparing M5 embryos with D3 embryos (comparison M5-D3).

Approximately 970 individual protein spots on three replicate gels were detected for each M5, ZEf and D3 embryo. These proteins were present for each type of sample. Among them, only 224 were significantly different (*P* < 0.05, ratio between normalized spot abundances of ≥ 2), with 169 and 91 in the M5-ZEf and M5-D3 comparisons, respectively (Table 2). Some proteins had significantly different abundances in both comparisons. Almost all of them were more abundant (8 proteins, Table 2) or less abundant (28 proteins, Table 2) in M5 embryos than in the other type of samples, indicating that M5 embryos were very different from ZEf and to a lesser extent from desiccated SEs. PCA, which clearly separated the samples according to the abundance of significant proteins (Supplementary Figure 1), indicated D3 had a medium composition between the M5 and ZEf proteomes in Dim 1 (64.5% of the explained variance). The proteins were identified by mass spectrometry. The level of abundance of each significant protein was compared according to the type of sample (Supplementary Table 4). It appeared that the most abundant proteins in M5 embryos in the comparison ‘M5-D3’ were also more abundant in the comparison ‘M5-ZEf’. Similarly, the proteins which became more abundant during SE desiccation (in D3) were also more abundant in ZEf than in M5 embryos.

**TABLE 2:** Distribution of 224 significant proteins identified in the two proteomic comparisons between cotyledonary somatic embryos matured for five weeks (M5) and fresh cotyledonary zygotic embryos (M5-ZE) or between M5 embryos before and after three weeks of desiccation (M5-D3), in the function of the overexpression ways.

			Comparison M5-ZE (169 sign.*^a^* proteins)	
			M5 < ZE*^b^*	M5 > ZE*^b^*
		**protein number*^c^***	116	53
**Comparison M5-D3 (91 sign.^a^. proteins)**	**M5 < D3*^b^***	54	28	0
	**M5 > D3*^b^***	37	1	8

*^a^sign.: significant; ^b^x > y: proteins more abundant in x than in y and vice versa, ^c^number of unique proteins identified in the two analyses.*

Functional annotation of the 224 proteins was performed according to the gene ontology classification system using homologous proteins in *Arabidopsis thaliana*. In this classification, a gene ontology (GO) term for molecular functions or biological processes is not attributed to every protein. In order to consider every protein, we manually adapted this classification by introducing two additional categories: “stored energy” and “desiccation protein”, corresponding to the parent molecular functions of the concerned proteins. The fold changes of protein abundance and molecular functions and biological processes assigned to the significant proteins are indicated in Supplementary Table 5.

**FIGURE 11:**
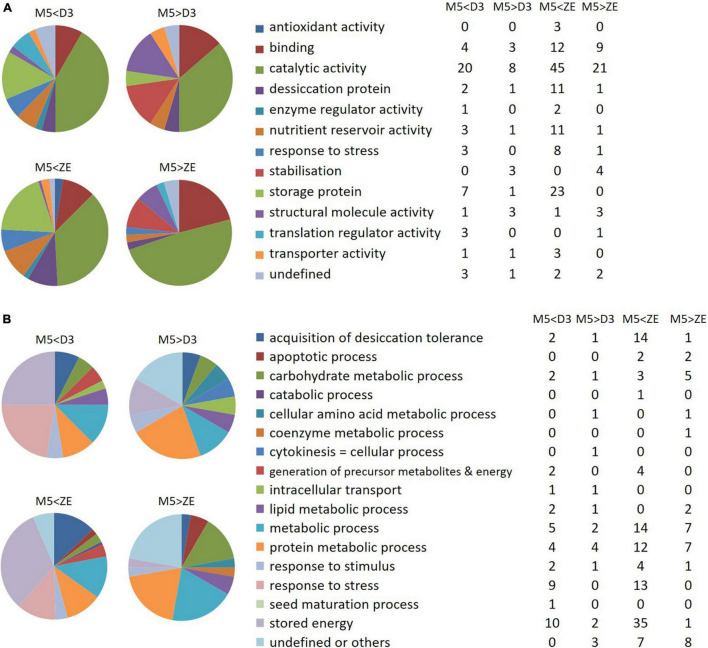
Gene ontology analysis of differentially expressed proteins. **(A)** Molecular function of the differentially abundant proteins between mature SEs (M5) and fresh ZEs or SEs after three weeks of desiccation (D3). **(B)** Biological processes modified by the differentially abundant proteins between the proteomes of M5 and fresh ZE, or of M5 and D3. On the left side of the figure, the colors represent the percentages of proteins involved in the indicated molecular function or biological process, whereas on the right side of the figure, the tables indicate the numbers of corresponding proteins. M5 > ZE and M5 > D3: proteins overexpressed in M5 in comparison to ZE or D3; M5 < ZE and M5 < D3: proteins overexpressed in ZE and D3, respectively.

Molecular functions of the proteins over- or under-expressed in ZEf were the same as those found in D3 (Figure 11A). Stress or desiccation response proteins, proteins with catalytic or nutrient reservoir activities, and storage proteins were amplified in D3 embryos and ZEf. Almost no molecular function was amplified in M5 embryos compared to samples D3 and ZEf. Only the function of stabilization was reduced in D3 and ZEf compared to M5 embryos.

The same biological processes were found to be activated in ZEf as in D3 embryos when compared to M5 embryos, according to the pattern observed at the scale of the modified molecular functions (Figure 11B).

Thus, the proteomes of ZEf and D3 embryos were more involved than that of M5 embryos in the “acquisition of desiccation tolerance“, “generation of precursor metabolites and energy”, “metabolic process”, “response to stress“, “response to stimulus” and “stored energy” processes. This last category included the most abundant proteins, which were cupin family storage proteins cruciferina and cruciferin 3. Only the biological process “protein metabolic process” was more active in ZEf than M5 embryos, whereas it was equivalent in M5 and D3 embryos.

## Discussion

For several conifers, including Norway spruce, it has been reported that conversion of embryos to plantlets can be improved by applying a desiccation period to aid reaching “physiological maturity” and positively influence germination (Stasolla and Yeung, 2003; Maruyama and Hosoi, 2012; Hazubska-Przybyl et al., 2015; Liao and Juan, 2015). In our previous experiments, embryos of the line AFO 541 cultivated without desiccation treatment were not able to develop radicles. 60–80% of embryos germinated after three weeks of desiccation, in contrast to only 20% of embryos that germinated without desiccation treatment. Non-completed emblings could not be transferred to *ex vitro* systems (Vondráková et al., 2013).

Our new analyses in the present study, using the same line AFO 541, provide a set of structural and biochemical parameters before (M5), after (D2-D3) or without (M8) desiccation treatment and show unambiguous effects of the desiccation phase in comparison with prolonged maturation. We demonstrated that the desiccation phase induces molecular and biological shifts that are not triggered by prolonged maturation. These modifications seem to correspond to the usual late maturation events that occur in most seeds (Angelovici et al., 2010; Leprince et al., 2017), including conifer seeds (Carrier et al., 1999; Silveira et al., 2004; Balbuena et al., 2011). Prolonged maturation resulted in callus formation on the surface of most embryos. The onset of callogenesis was detected after only six weeks of maturation (Eliášová et al., 2018). Callus never formed on desiccated embryos. Our comparison of SEs and ZEs showed some similarities (to a certain extent) in storage proteins and non-structural carbohydrates changes. The proteomic analysis confirmed these similarities. Biological processes activated in D3 embryos were the same as those activated in ZEf when both were compared to M5 embryos. These changes were consistent with data from previous studies (e.g., Hazubska-Przybyl et al., 2015; Liao and Juan, 2015). In contrast, the results of the phytohormone analysis indicated quantitative differences between SEs and ZEd.

### Modifications of Storage Compound Levels

Storage reserves may help an embryo to tolerate desiccation by increasing the dry weight (Attree et al., 1995; Pérez et al., 2020). They are used as sources of energy and amino acids during seedling germination and growth until autotrophy is acquired. Their quantity (Kong et al., 1999) and quality are important variables dictating the future vigor of the seedling. Storage reserves are gradually produced to varying degrees at different stages of development. Joy et al. (1991) reported that starch accumulated first, followed by lipids and proteins during later maturation stages of white spruce. Compared to mature ZEd of white spruce or ZEf of maritime pine (Tereso et al., 2007), SEs of both these species accumulated more starch. However, the cotyledonary ZEf of maritime pine showed numerous starch grains, in contrast to the ZEd of white spruce. In Norway spruce, we detected a high amount of starch grains in M5 embryos, which decreased during the first two weeks of prolonged maturation. The comparison we made with ZEf at the histological level (Figure 3) revealed an accumulation of starch grains comparable to that of M5 embryos. Longer cultivation of SEs on the maturation medium resulted in significantly higher starch accumulation, which could be ascribed to ectopic starch grain accumulation in M8 embryos that often suffered from morphological abnormalities and callogenesis. On the contrary, the desiccation treatment caused a substantial reduction in the starch content, which approached a very low level in ZEd. The biochemical determination generally confirmed our analysis based on histochemical evidence. These results indicate that starch represents a transient energy source in both SEs and ZEs. Most importantly, they imply the preparation of the embryos in a dry state, where the starch content is low after conversion into sugars such as sucrose and RFOs, which helps to prevent lethal effects of water stress (Yathisha et al., 2020).

In conifers, proteins make an important contribution to the energy reserves of seeds and embryos. Our results showed that the protein content at the end of maturation was very similar to that already reported in *P. abies* by Businge et al. (2013). However, it was lower than in ZEf, indicating that the somatic embryogenesis protocol does not provide embryos with protein energy reserves equivalent to those present in ZEf. This phenomenon has been widely observed in the somatic embryogenesis of conifers (Klimaszewska et al., 2004; Businge et al., 2013). Desiccation, during which SEs are no longer provided with nutrients, causes a small drop in protein content in embryos, whose maintenance of respiration and minimum metabolism consume storage compounds, as shown by the degradation of PSVs in D3 embryos (Figure 2). Although a slight decrease in protein content was also observed in SEs whose maturation was extended to eight weeks (samples M8), this must indicate another phenomenon. We interpreted the decline as going beyond the late maturation stage, during which energy reserves accumulate (Leprince et al., 2017) and engaging in the germination process, as has already been described for *Pinus sylvestris* SEs (Lelu-Walter et al., 2008). Qualitatively, the same protein profiles were obtained for mature and desiccated SEs as for ZEs. More intense bands corresponding to reserve proteins have already been identified in *P. abies* (Hakman et al., 1990; Businge et al., 2013), although they were not localized on the gel, unlike in our study. These corresponded to ubiquitous storage proteins (vicilin- and legumin-like globulins) in plants that have already been identified for many conifers, e.g., maritime pine (Morel et al., 2014) and larch (Teyssier et al., 2014). Lara-Chavez et al. (2012) observed an increase in transcripts encoding the legumin- and vicilin-like proteins at the end of maturation, but these proteins may be expressed only later if the conditions are favorable (Leprince et al., 2017). This is consistent with our findings of the increased storage proteins production in D3 embryos compared to M5.

### Soluble Carbohydrate Profile of Desiccated Somatic Embryos Resembles the Profile of Zygotic Embryos

M5 embryos have a typical carbohydrate spectrum with a high sucrose:hexose ratio. A very similar robust pattern was described earlier in the same line (Lipavska et al., 2000) and also in another highly embryogenic *P. abies* line (Hudec et al., 2016) or *P. glauca* and *P. mariana* lines (Iraqi and Tremblay, 2001). In contrast to prolonged maturation, during desiccation, remarkable changes in the amount and spectrum of soluble carbohydrates were induced in SEs. Most importantly, desiccation induced the synthesis of RFOs, which have been reported to be a substantial component of mature conifer ZEs (e.g., Downie and Bewley, 2000; Gösslová et al., 2001; Konradova et al., 2003; Pullman and Buchanan, 2008). During somatic embryogenesis, it has been shown in some species that RFOs accumulate as early as during maturation (Carpenter et al., 2000; Morel et al., 2014). In Norway spruce SEs, their accumulation is associated with the effect of post-maturation treatments, e.g., fast desiccation (Bomal et al., 2002), high humidity desiccation (Konradova et al., 2003; Kubes et al., 2014; Hudec et al., 2016) or cold treatment (Konradova et al., 2003). However, it remains controversial whether the presence of RFOs is responsible for improving embryo germination. Several reports have indicated that levels of RFOs are closely linked to the degree of tolerance to stress. Carpenter et al. (2000) suggested that an appropriate marker for the full maturity of SEs of the genus Pinus is the level of oligosaccharides of RFOs and dehydrins, with increased RFO levels resulting in improved germination. Businge et al. (2013) attributed improved germination of *P. abies* SEs to high levels of sucrose, raffinose and LEA proteins, which promoted the acquisition of desiccation tolerance. Bomal et al. (2002) stated that a high sucrose content and occurrence of raffinose were in agreement with the development of tolerance to fast desiccation in *P. mariana* SEs. The protective action of sucrose has been attributed to its capacity to stabilize proteins and membranes, probably by replacing their hydration coatings (Crowe et al., 1992). Raffinose increases the availability of sucrose by preventing its crystallization, and thus enhances the protective action of sucrose (Koster and Leopold, 1988; Oliver et al., 1998). For this reason, it is often suggested that the RFO:sucrose (R/S) ratio is more critical for desiccation protection than absolute carbohydrate levels (Sauter and van Cleve, 1991; Xiao and Koster, 2001). The optimal RFO:sucrose ratio reported to maintain the glassy state is 1:5.7 (approx. 0.18) (Koster, 1991; Koster and Bryant, 2005), but a much lower ratio of 1:20 (0.05) has been shown to be sufficient (Lin and Huang, 1994). Here, a functional complex of sucrose and RFOs appears to be used as well (0.18 for D2 and 0.37 for D3 embryos).

Another possible action of RFOs during desiccation is a reduction in the availability of monosaccharides due to the synthesis of RFOs, leading to the suppression of respiration, and thus metabolic activity of the seed (Leprince et al., 1992; Pammenter and Berjak, 1999). Finally, they may also play a role as quenchers of reactive oxygen species formed during dehydration (reviewed by Keunen et al., 2013).

Raffinose family oligosaccharide biosynthesis begins with the reversible transfer of a galactosyl residue from galactinol as the most common donor to a sucrose molecule to form raffinose (Keller and Pharr, 1996; Peterbauer and Richter, 2001). Thus, monitored levels of galactinol in SEs compared to those in ZEd might indicate a slightly different coordination/balance of carbon distribution into compounds closely related to RFO synthesis. Galactinol probably provides a similar protective function for embryos, and thus complements the protective function of this group of substances.

The carbohydrate status in desiccated SEs of Norway spruce corresponded better with that of ZEd, as the endogenous soluble carbohydrate spectrum of these dormant, winter-harvested ZEs was characterized by a comparable amount of sucrose, high proportion of RFOs and only low amounts of glucose, fructose and inositol. The total level of carbohydrates per unit DW was significantly lower in ZEs compared to SEs (in agreement with Konradova et al., 2003).

Although the level of RFOs in SEs after desiccation treatment did not reach that in ZEs, it was still enough to secure the multiple functions mentioned above. Similarly, lower R/S ratios found in SEs compared to their zygotic counterparts exceeded the R/S value considered sufficient for the protective function of RFOs.

### Modifications of Phytohormone Content

Substantial differences were found in the content of analyzed phytohormones (ABA, auxins and CKs) in SEs during prolonged maturation and desiccation. Significantly lower levels of phytohormones were detected in ZEd, with profiles of particular derivatives mostly similar to those of SEs.

Abscisic acid determines conifer SE maturation and germination. Its application induces the start of SE maturation and is essential for successful embryo development (e.g., von Arnold and Hakman, 1988; von Arnold et al., 1996; Filonova et al., 2000; von Aderkas et al., 2002). Although the whole maturation process has been shown to continue on medium weekly supplemented with a high concentration of ABA, a reduction of endogenous ABA content (necessary for optimal germination) begins already during the second part of maturation (Vondrakova et al., 2018). Our present data show that ABA reduction further continues during prolonged maturation. However, no substantial influence was evident on the composition of ABA-type derivatives in M7 and M8 embryos.

A decrease in ABA levels is usually considered as the main effect of desiccation treatment (Kermode et al., 1989; Find, 1997). Lower levels of ABA are necessary for successful germination, e.g., in seeds and/or embryos of *Picea glauca* and *Picea morrisonicola* (Liu et al., 2015; Liao and Juan, 2015). We detected a larger decrease of free ABA concentration in D2 and D3 embryos when compared to M3 and M5 embryos (Vondrakova et al., 2018) as well as M7 and M8 embryos (as demonstrated here).

Moreover, desiccation treatment also induced a reduction in the content of ABA derivatives PA and DPA. The decrease in concentrations of ABA, PA, and DPA in desiccated embryos was mainly linked to the change of cultivation conditions, i.e., the absence of exogenous ABA. ABA-GE (ABA storage conjugate) was present in maturated as well as in desiccated embryos. Relatively constant levels of ABA-GE have been observed during seed development of Douglas-fir (Chiwocha and von Aderkas, 2002).

During maturation, comparable concentrations of total auxins were found in M5, M7 and M8 embryos. It was previously demonstrated that maximal auxin concentrations during maturation correlate with the polarization of SEs (Vondrakova et al., 2018). In this step, the IAA-amino acid conjugates IAA-Asp and IAA-Glu are the main auxin forms. Similarly, a high level of IAA-Asp has been detected during SE and ZE maturation in Douglas-fir and larch (von Aderkas et al., 2001; Chiwocha and von Aderkas, 2002). The content of auxins decreases from the third to the fifth week of maturation and the participation of IAA-Asp and IAA-Glu is minimal in M5 embryos (Vondrakova et al., 2018). As shown here, IAA was the main auxin in M5 embryos, followed by the non-indole weak auxin PAA. During prolonged maturation, the share of IAA decreased, whereas that of IAA-Asp increased. Despite these changes in the proportion of particular auxin derivatives, the total content of auxins remained at a low and relatively constant level. This may be the reason for the rooting inability of SEs because auxins regulate the root pole formation and rooting of embryos (Brunoni et al., 2019). In our experimental system, similar concentrations of auxins were found in M5, M7, M8 and D2 embryos, and an increase in auxin levels was detected just before germination (D3). The main difference between matured and desiccated embryos was in the proportion of different auxins. Although IAA-Asp was prevalent in M7 and M8 embryos, Ox-IAA was predominant in desiccated embryos. Oxidation of IAA is an important step to control auxin concentrations in angiosperms and, along with IAA conjugation, to respond to perturbation of IAA homeostasis (Brunoni et al., 2020). Our finding of Ox-IAA in desiccated SEs and ZEd is quite surprising as Brunoni et al. (2020) have shown that conjugation predominates over oxidation in maintaining IAA homeostasis in Norway spruce seedlings.

Endogenous levels of CKs have been shown to be rather low during maturation, but their diversity is high (Vondrakova et al., 2018). As shown here, the proportion and content of CKs remained unchanged throughout prolonged maturation (M7, M8) compared to M5 embryos. During prolonged maturation, the CK pool was represented mainly by *cis*Z- and DHZ-types, while iP- and *trans*Z-types occurred in significantly lower concentrations. This finding corresponds well with the results of Quesnelle and Emery (2007), who found that *cis*-zeatins were the primary CK forms during pea embryogenesis, whereas corresponding *trans-*isomers were only minor constituents.

A large increase in concentration was found in desiccated embryos for all CK forms except iP-types, mainly due to the enhanced accumulation of *O*-glucosides (predominantly DHZROG and *cis*ZROG). Involvement of these *O*-glucosides was demonstrated in our previous work during the desiccation and germination of Norway spruce SEs (Vondrakova et al., 2018). Cytokinin *O*-glucosides are generally regarded to accumulate in mature and senescing tissues (Letham and Palni, 1983), and the *O*-glucosylation is thought to be reversible since ß-glucosidases are capable of hydrolyzing them in plants (Brzobohatý et al., 1993). *O*-glucosylation represents a rapid and efficient mechanism of CK inactivation, but the role of *O*-glucosides in plant biology remains unclear (Záveská-Drábková et al., 2021). Unfortunately, no other studies have demonstrated a potential role of cytokinin *O*-glucosides in SE development, since only fundamental CKs (free bases and ribosides) and other basic auxins and ABA derivatives (ABA, ABA-GE, IAA, IAA-Asp) were analyzed in somatic embryogenesis until recently due to limitations in the available methodology (Jourdain et al., 1997; Vágner et al., 1998; von Aderkas et al., 2001; Zhou et al., 2017).

In all our analyses, extremely low concentrations of phytohormones were found in ZEd. Despite this, the hormonal spectra mostly corresponded with those in M5 embryos, especially in the case of ABA derivatives and auxins. However, in contrast to SEs, ZEd also contained cytokinin *N*-glucosides (*N9*- but not *N7*-glucosides), apparently irreversible forms widely distributed throughout the plant kingdom (Pokorná et al., 2021). Very low concentrations of phytohormones in Norway spruce ZEd suggest a possible correlation between the minimum WC in these winter seeds and their low biochemical activity, which seems to be related to the deep dormancy of Norway spruce seeds during the winter season.

### Modifications of the Proteome of Desiccated Somatic Embryos Make Them Closer to Zygotic Embryos

Comparative proteomic analyses revealed significantly differentially accumulated proteins between the compared proteomes. Biological processes modified by desiccation are discussed in the light of functional classes of GO. The processes most represented by the identified significant proteins were “Metabolic process” - “Stored energy”, “Acquisition of desiccation tolerance”, “Response to stimulus”, and “Response to stress”. They corresponded to those often amplified during the late maturation of various conifer seeds or SEs (Stasolla et al., 2003; Stasolla et al., 2004b; Silveira et al., 2008) or in comparison of late somatic and zygotic maturation (Balbuena et al., 2009; Lara-Chavez et al., 2012; Morel et al., 2014; Jing et al., 2017).

As in many studies, we did not observe overexpression of proteins involved in photosynthesis which are activated after germination. However, this has been investigated in a recent study analyzing the mechanisms taking place during partial desiccation of SEs of *Pinus asperata* (Jing et al., 2017). In our study, the changes in proteomic profile observed between D3 and M5 embryos were very similar to those between ZEf and M5 embryos, with most of the modified biological processes being amplified. This suggests that (1) desiccation causes protein reprogramming, resulting in a proteome approaching that of ZEf, as shown by the carbohydrate and auxin assays, and (2) desiccation treatment induces some biological processes without inhibiting any of those expressed in M5 embryos. Nevertheless, the number of significant proteins, as well as the abundance ratios modified by desiccation, were lower than those in ZEf. Therefore, even if desiccation induced reprogramming of the proteome of M5 embryos toward ZEf (Fait et al., 2006; Angelovici et al., 2010), D3 was not yet fully equivalent to ZEf (Supplementary Figure 1). This was confirmed by the multivariate analysis based on all biochemical variables measured (Figure 10).

The main protein functional classes amplified after desiccation, as well as RFO synthesis and modification of phytohormone profiles observed in this study, participate in the characteristic events of the late maturation phase of embryos, i.e., activation of storage metabolism of energy reserves, acquisition of desiccation tolerance and development of stress response systems.

#### Activation of Storage Metabolism of Energy Reserves

The accumulation of storage reserves before the quiescent state of embryos is necessary to support the germination and growth of the seedlings before their energy autonomy. In conifers, these reserves are stored mainly in protein and lipid forms. Both D3 embryos and ZEf showed overexpression of storage proteins but without abundance modification of the enzymes involved in their synthesis. Thus, we deduced that their syntheses took place earlier before stages D3 and ZEf. Storage proteins are major sources of nitrogen and carbon during subsequent seed germination and early seedling growth. The presence of these proteins in various isoforms is due to the multigenic character of these proteins grouped into families (Li et al., 2007). The identified cruciferin and cruciferina, members of the cupin superfamily, are respectively vicilin- and legumin-like protein globulins. Among the storage proteins of conifers, they represent the majority of storage forms (Hakman et al., 1990; Flinn et al., 1993; Lelu-Walter et al., 2008; Teyssier et al., 2011; Morel et al., 2014). We also detected an albumin 2S storage protein on the retard gel (Figure 4), the abundance of which was not modified by desiccation. However, the synthesis of energy reserves may begin before the acquisition of tolerance to desiccation (Marques et al., 2019); we deduced that the synthesis of 2S albumin occurred prior to the M5 stage.

Lipids are another source of energy reserve during the germination of conifer embryos but also for their respiratory activities during the late maturation phase (Leprince et al., 2017). However, we did not detect overexpression of the proteins involved in their synthesis. Although the sequence of storage deposition processes occurring at the end of embryogenesis is often species-specific, the synthesis of storage lipids often takes place prior to or simultaneously with the synthesis of storage proteins (Righetti et al., 2015; Leprince et al., 2017). This has been shown for *P. abies*, in which lipid synthesis is maximal after 4 weeks of maturation (Grigová et al., 2007).

In SEs (Vestman et al., 2011; Jing et al., 2017), as in orthodox seeds (Ntuli et al., 2011), the end of maturation is associated with higher activities of the systems involved in stress defense (stress-related proteins, RFOs, secondary metabolites) in prevision of the increased level of oxidation of cells during dehydration. Also, the correlation between the amplified presence of stress-related proteins and the acquisition of desiccation tolerance has been demonstrated in many studies and their synthesis directly contributes to this acquisition (Stasolla et al., 2004b; Finnie et al., 2006; Silveira et al., 2008; Sghaier et al., 2009; Shi et al., 2010).

#### Acquisition of Desiccation Tolerance

Desiccation tolerance is a multifactorial trait that involves the protection of macromolecules and membranes from the deleterious effects of water removal. The best-known proteins involved in this protection are late embryogenesis abundant proteins (LEA) and heat shock proteins (HSPs) (Stasolla et al., 2003; Personat et al., 2014). Only a few LEA proteins were identified among the proteins whose abundance was modulated by desiccation in our study. These were Em-like protein GEA6, belonging to LEA group 1, late embryogenesis abundant domain-containing protein and LEA 31. All of these proteins were more abundant in D3 than in M5 embryos. This also applied to all LEAs identified in the M5/ZEf analysis, which were then more numerous in ZEf. LEA proteins are sequentially synthesized during embryo development, probably related to their very diverse functions during late maturation and germination (Chatelain et al., 2012), with maximal abundance at the late maturation stage (Leprince et al., 2017). In conifers, many studies have shown the presence of their transcripts from the middle of maturation (Stasolla et al., 2003; Vales et al., 2007; Silveira et al., 2008) or the presence of the proteins at the end of the maturation (Silveira et al., 2008; Businge et al., 2013; Morel et al., 2014). Furthermore, many LEAs may already be synthesized prior to the M5 stage, and therefore cannot be revealed by a comparative proteomic study.

#### Development of Stress Response Systems

Stress-related proteins, generally present in the late maturation stage, are classified as stress- or stimulus-response proteins. These proteins, which stabilize molecular structures (chaperones, HSPs), maintain homeostasis and neutralize deleterious reactive oxygen species (ROS) (e.g., catalase, superoxide dismutase), participate in different stages of embryo development (Fehér et al., 2003). Their expression has been shown to be amplified after a post-maturation treatment inducing desiccation tolerance and increased germination capacity in *Pinus asperata* SEs (Jing et al., 2017). This was not the case in D3 embryos, although the treatment did improve the germination ability, as seen in our previous study (Vondráková et al., 2013). It is important to dissociate the aptitudes of embryos for desiccation from the aptitudes for germination. Since HSPs are involved in the maintenance of membrane and molecular structures in cells in low WC, this protection is not essential for late-maturing embryos for which the water status remains normal.

Anticipation of the deleterious oxidative damage inherent in the desiccation of embryos involves the accumulation of antioxidant enzymes that can neutralize them. In this study, several such proteins were identified (glutathione peroxidase 6, glutathione transferase-29, glyoxalase, lipocalin-1, and oxidoreductase), which were all up-regulated in D3 and ZEf. In the latter, the panel of proteins was more extensive since it was complemented by superoxide dismutase, peroxiredoxin type 2 and 1-cysteine peroxiredoxin 1. Whereas only the latter has been shown to be expressed during the acquisition of dehydration tolerance (Leprince and Buitink, 2010), the others may be active during other periods of plant development characterized by a high content of hydrogen peroxide, such as germination or early embryogenesis (Huang et al., 2012; Jing et al., 2017).

To summarize, unlike prolonged maturation, the post-maturation desiccation treatment triggered (i) a significant decrease in ABA content and shift in ABA catabolism toward the accumulation of storage form ABA-GE, (ii) a decrease in the content of starch, (iii) accumulation of RFOs, storage proteins, LEA and stress-related proteins, and (iv) activation of antioxidative systems. These modifications could be markers of the acquisition of desiccation tolerance. Given their simultaneous occurrence at the late maturation stage, it is reasonable to assume the entry of mature SEs into this process.

## Conclusion

The present work revealed that SEs were modified during desiccation, i.e., post-maturation treatment at high relative humidity, on all levels tested. The accumulation of compounds (RFOs, LEA) that are believed to protect cell structures from the consequences of desiccation strongly suggests that SEs approach a state of tolerance to drying without a marked decrease in water content. The control realized by prolonging the maturation for the same time as the desiccation treatment applied in parallel confirmed that the observed metabolic and physiological shifts were indeed the consequence of the applied treatment. The biological process changes observed in the treated SEs were very similar to those occurring in ZEs during the late phase of maturation of orthodox seeds before the winter season. We suggest that they may be important for further embryo development and contribute to successful germination.

## Data Availability Statement

The original contributions presented in the study are included in the article/Supplementary Material; further inquiries can be directed to the corresponding author.

## Author Contributions

KE, ZV, CT, and M-AL-W conceived and planned the experiments. ZV and LF prepared all the plant material. KE carried out the histological analyses. HK performed the carbohydrate content quantification. PD and VM performed phytohormone analyzes. CT and A-ML carried out the proteomic analysis. ZV and CT carried out the statistical analysis. KE, ZV, CT, HK, VM, and M-AL-W wrote and reviewed the manuscript. All authors discussed the results and contributed to the final manuscript.

## Conflict of Interest

The authors declare that the research was conducted in the absence of any commercial or financial relationships that could be construed as a potential conflict of interest.

## Publisher’s Note

All claims expressed in this article are solely those of the authors and do not necessarily represent those of their affiliated organizations, or those of the publisher, the editors and the reviewers. Any product that may be evaluated in this article, or claim that may be made by its manufacturer, is not guaranteed or endorsed by the publisher.

## Footnotes

1
https://conekt.sbs.ntu.edu.sg/species/view/9

2
https://www.arabidopsis.org/

3
http://pantherdb.org/

## Abbreviations

D2, D3 embryos: embryos desiccated for 2 or 3 weeks
HSP: heat shock protein
LEA: late embryogenesis abundant protein
M5 embryos: mature embryos after 5 weeks of maturation
M7, M8 embryos: embryos after 2 or 3 weeks of prolonged maturation
RFOs: raffinose family oligosaccharides
SE: somatic embryo
ZE: zygotic embryo
ZEf: fresh zygotic embryos
ZEd: dry zygotic embryos
ABA: abscisic acid
ABA-GE: ABA-glucose ester
*cis*Z9G: *cis*-zeatin 9-glucoside
*cis*ZOG: *cis*-zeatin *O*-glucoside
*cis*ZR: *cis*-zeatin 9-riboside
*cis*ZRMP: *cis*-zeatin 9-riboside-5′-monophosphate
*cis*ZROG: *cis*-zeatin 9-riboside *O*-glucoside
DHZ: dihydrozeatin
DHZR: dihydrozeatin 9-riboside
DHZRMP: dihydrozeatin 9-riboside-5′-monophosphate
DHZROG: dihydrozeatin 9-riboside *O*-glucoside
DHZ9G: dihydrozeatin 9-glucoside
DPA: dihydrophaseic acid
IAA: indole-3-acetic acid
IAA-Asp: IAA-aspartate
IAA-Glu: IAA-glutamate
iP: *N*^6^-(Δ^2^-isopentenyl) adenine
iPR: *N*^6^-(Δ^2^-isopentenyl) adenosine
iPRMP: *N*^6^-(Δ^2^-isopentenyl) adenosine-5′-monophosphate
NeoPA: neophaseic acid
9OH-ABA: 9-hydroxy-ABA
OxIAA: oxo-IAA
PA: phaseic acid
PAA: phenylacetic acid
*trans*Z: *trans*-zeatin
*trans*ZOG: *trans*-zeatin *O*-glucoside
*trans*ZR: *trans*-zeatin 9-riboside
*trans*ZRMP: *trans*-zeatin 9-riboside-5′-monophosphate
*trans*ZROG: *trans*-zeatin 9-riboside *O*-glucoside.
