# Supplementary material for: Desiccation as a Post-maturation Treatment Helps Complete Maturation of Norway Spruce Somatic Embryos: Carbohydrates, Phytohormones and Proteomic Status
Source: Front Plant Sci. 2022 Feb 14;13:823617. doi: 10.3389/fpls.2022.823617 (PMC8882965; doi:10.3389/fpls.2022.823617)

**Supplementary Figure 1: PCA of somatic embryos maturing during five weeks (M5) or desiccated during three weeks (D3), and fresh zygotic embryos (ZE) according to the significant protein spots** obtained in proteomic 2D analysis, in the factorial plan Dim 1 - Dim 2.


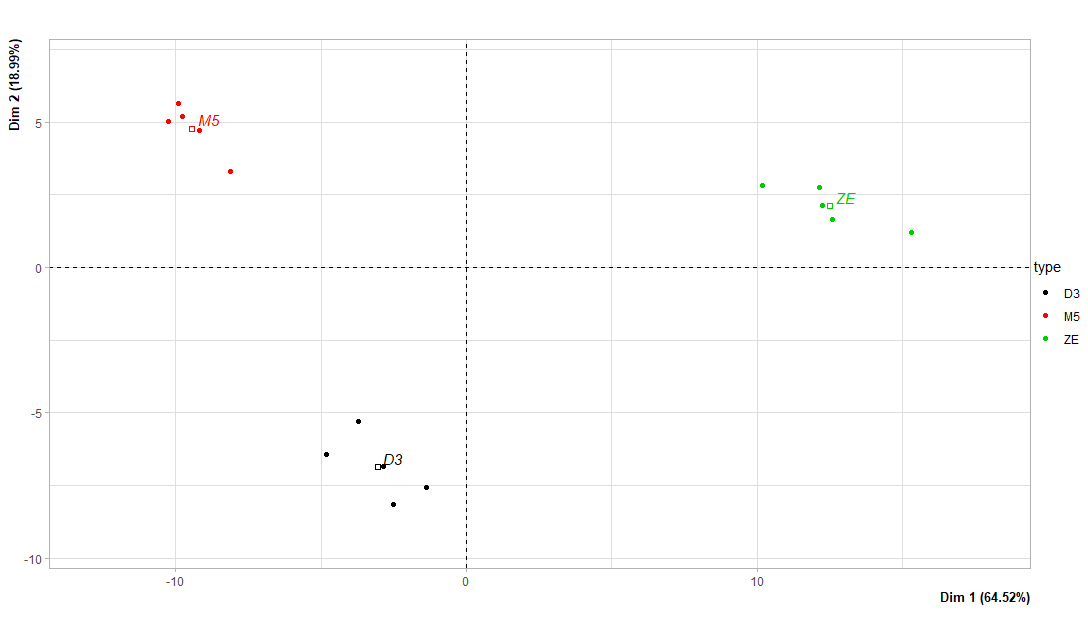

Supplement: Supplementary file 1 [file Table_1.DOCX]
